# A new classifier constructed with platelet features for malignant and benign pulmonary nodules based on prospective real-world data

**DOI:** 10.7150/jca.67428

**Published:** 2022-05-09

**Authors:** Ruiling Zu, Lin Wu, Rong Zhou, Xiaoxia wen, Bangrong Cao, Shan Liu, Guishu Yang, Ping Leng, Yan Li, Li Zhang, Xiaoyu Song, Yao Deng, Kaijiong Zhang, Chang Liu, Yuping Li, Jian Huang, Dongsheng Wang, Guiquan zhu, Huaichao Luo

**Affiliations:** 1 Department of clinical laboratory, Sichuan Cancer Hospital & Institute, Sichuan Cancer Center, School of Medicine, University of Electronic Science and Technology of China, Chengdu, Sichuan, China; 2 Chiang Mai University College of International College of Digital Innovation, Chiang Mai, Thailand; 3 Department of Clinical Laboratory, Sichuan Academy of Medical Sciences & Sichuan provincial People's Hospital, Chengdu, China; 4 Chengdu University of Traditional Chinese Medicine, Chengdu, Sichuan, China; 5 Radiation Oncology Key Laboratory of Sichuan Province, Sichuan Cancer Hospital & Institute, Sichuan Cancer Center, School of Medicine, University of Electronic Science and Technology of China, Chengdu, China; 6 State Key Laboratory of Oral Diseases, National Clinical Research Centre for Oral Diseases, Department of Head and Neck Oncology, West China Hospital of Stomatology, Sichuan University, Chengdu, China; 7 Center for Informational Biology, School of Life Science and Technology, University of Electronic Science and Technology of China, Chengdu, Sichuan, China

**Keywords:** lung cancer, pulmonary nodules, platelets, diagnosis, XGBoost, clinical laboratory

## Abstract

**Objectives:** As the pulmonary nodules were hard to be discriminated as benignancy or malignancy only based on imageology, a prospective and observational real-world research was devoted to develop and validate a predictive model for managing the diagnostic challenge.

**Methods:** This study started in 2018, and a predictive model was constructed using eXtreme Gradient Boosting (XGBoost) based on computed tomographic, clinical, and platelet data of all the eligible patients. And the model was evaluated and compared with other common models using ROC curves, continuous net reclassification improvement (NRI), integrated discrimination improvement (IDI), and net benefit (NB). Subsequently, the model was validated in an external cohort.

**Results:** The development group included 419 participants, while there were 62 participants in the external validation cohort. The most accurate XGBoost model called SCHC model including age, platelet counts in platelet rich plasma samples (pPLT), plateletcrit in platelet rich plasma samples (pPCT), nodule size, and plateletcrit in whole blood samples (bPCT). In the development group, the SCHC model performed well in whole group and subgroups. Compared with VA, MC, BU model, the SCHC model had a significant improvement in reclassification as assessed by the NRI and IDI, and could bring the patients more benefits. For the external validation, the model performed not as well. The algorithm of SCHC, VA, MC, and BU model were first integrated using a web tool (http://i.uestc.edu.cn/SCHC).

**Conclusions:** In this study, a platelet feature-based model could facilitate the discrimination of early-stage malignancy from benignancy patients, to ensure accurate diagnosis and optimal management. This research also indicated that common laboratory results also had the potential in diagnosing cancers.

## Introduction

All over the world, lung cancer has become the most frequent cancer and cause of cancer death. According to the report of Chinese cancer registration in China, there were 733.3 thousands of new lung cancer cases in 2015, which exceeded the number in 2007. The reports also revealed that the 5-year survival of lung cancer in 2012-2015 was 23.8% and in 2009-2011 was 20.3% 1, 2. Low-dose computed tomography (LDCT) has been accepted as an effective screening method in high-risk individuals to reduce lung cancer mortality 3, 4. The limits of LDCT screening included the inappropriate detection of lung nodules, for their high possibilities of being early lung cancers. Pathologically, those equivocal nodules could be only confirmed by invasive biopsy or postoperative biopsy, while a part of nodules were determined to be benign 3, 4. Therefore, some researchers were devoted to construct clinical models or liquid biopsies increasing the appropriate detection of LDCT.

The clinical models usually combine LDCT-detected nodule characteristics with clinical data of patients to estimate the probabilities of malignancy in pulmonary nodules, such as VA, MC and BU model 5-8. Those diagnostic models partly improved the diagnostic value of LDCT, but were still hard to distinguish benign from malignant nodules 9, 10. An accurate and practical model could indicate the probability that the equivocal nodule was malignant or benign and would reduce the misdiagnosis in screening programs.

Routine blood test (RBT) is a low-cost test including more than dozens of features and revealing the complications of individuals, which can be characterized as an easy and basic liquid biopsy and provides a large data for researches 11. In our previous research, we identified that platelet features based on RBT had potential to diagnose lung cancer, which meant the platelet features could complement LDCT to distinguish benign nodules from malignant nodules 12. In this research, we collected real-world data to construct a diagnostic model combining platelet features with clinical and LDCT information, which could be used to discriminate malignancy from benign nodules, and to expand the treatment options. The model was also presented firstly as a web tool.

## 2. Material and Methods

This prospective, observational and multi-center real-world study was started from 2018 until now in Sichuan Cancer Hospital and Sichuan Provincial People's Hospital. The data of development group were collected consecutively between January 2018 and December 2020 in Sichuan Cancer Hospital. The new external validation data were collected between March 2021 and April 2021 in Sichuan Provincial People's Hospital. The study was approved by the medical ethical committee of Sichuan Cancer Hospital (SCCHEC-02-2018-031, SCCHEC-02-2020-043), and Sichuan Provincial People's Hospital (2021-NO.404).

### 2.1 Study Design

**Figure 1:**
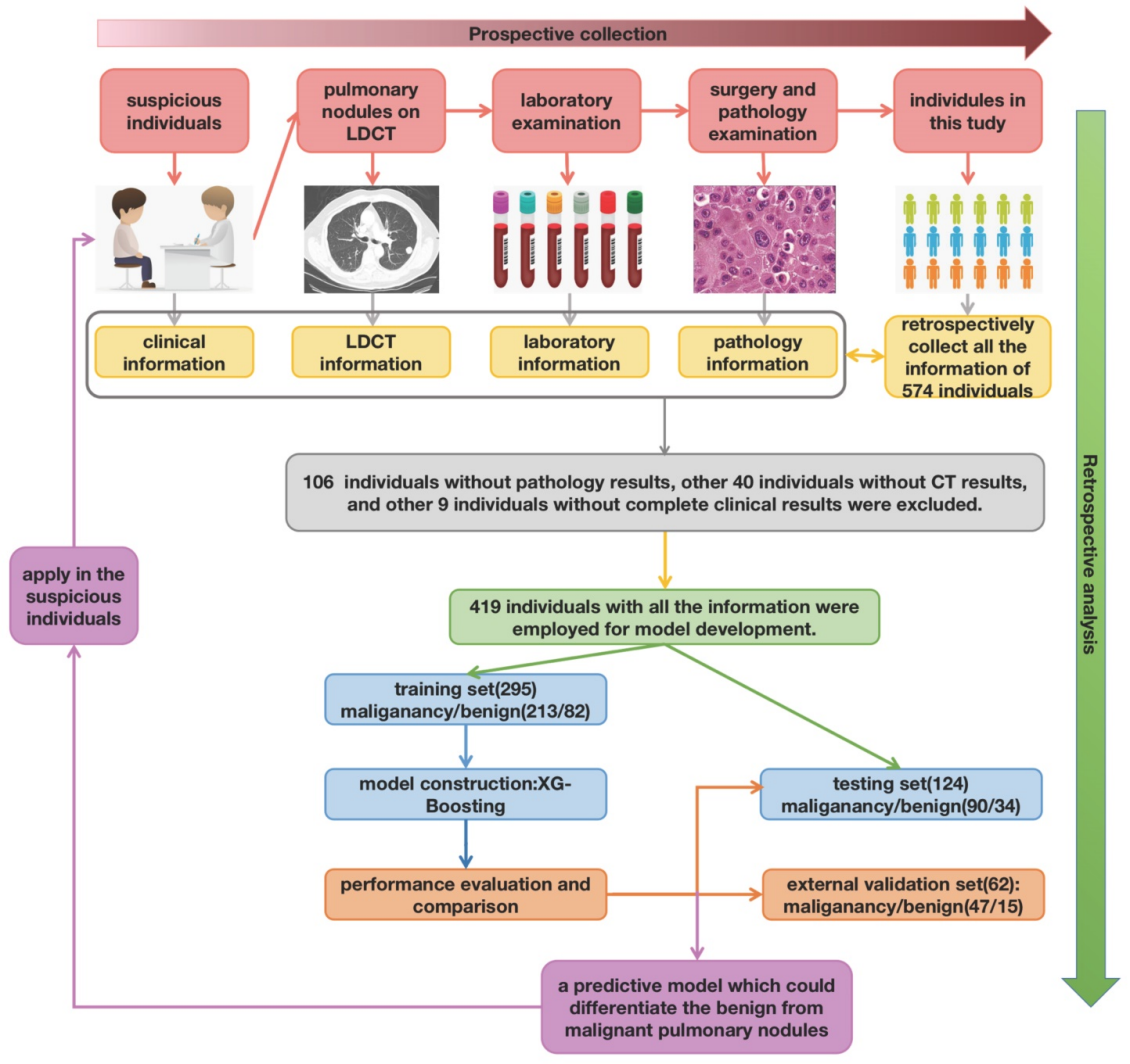
The work-flow chart of this study. Primarily, there were total of 574 individuals enrolled in this research. After retrospectively analyzed, 106 individuals without pathology results, other 40 individuals without CT results, and other 9 without complete clinical information were excluded.

The work-flow chart of this study was shown in Figure 1. The individuals consecutively diagnosed with a first occurrence of pulmonary nodules were enrolled in this study. Patients should be observed for the duration of the diagnosis and therapy line (up to the day the patient discharged from hospital). The blood samples were collected and determined at the same time participants admitted hospital 12. The pathological examination of resected tissue was finished after surgery or biopsy. The clinical information (age, gender, smoking history, anamnesis, family history, personal history), LDCT information (nodule size, number, characteristic, position), pathology information (pathological type, pathological stage), and laboratory information (platelet features in whole blood samples and platelet rich plasma samples) were captured and summarized.

In the development group, seventy percent of all participants were enrolled in the training cohort, while thirty percent of participants were enrolled in the testing cohort randomly. The diagnostic model achieved in training cohort, and validated in the testing and external cohort. Then the model was compared with other common models in the whole development group.

### 2.2 Study Participants

The development and external validation of the model based on the data of patients as follows. All the patients whose pulmonary nodules were first found in LDCT, and accepted the surgery treatment or histologic examination in the two hospitals were enrolled. According to the pathological results, the patients were divided into two groups: the benign group (patients with benign nodules) and the malignant group (patients with malignant nodules). The histological type and TNM staging were based on the guidelines of Chinese society of clinical oncology (2019 version) 34. All the LDCT information, pathology information, and laboratory information were also recorded.

And the exclusion criteria were as followed: 1) patients who were not confirmed by the surgery or biopsy; 2) patients with other cancers and acute inflammation; 3) patients who had received the medicine influencing the platelets such as aspirin; 4) patients who received platelet or blood transfusion in half of a month; 5) patients without complete clinical and laboratory data.

### 2.3 Laboratory Measures

In Sichuan Cancer Hospital, the pulmonary nodules were detected by SIEMENS Definition Flash CT (SIEMENS Healthineers Co., Ltd, Erlangen, GER) and PHILIPS Brilliance iCT (Koninklijke Philips N.V., Amsterdam, NED). As previously, the fresh whole blood samples were collected to detect platelet features and separate the platelet rich plasma (PRP) from nucleated blood cells by a 20-minute 120×g centrifugation step (Co., Ltd. Shuke, Chengdu, Sichuan, CHN). Meanwhile, the platelet features in the PRP samples were detected. The platelet features in whole blood samples and PRP samples were detected with a Mindray cell counter BC-6600 (Mindray Bio-Medical Electronics Co., Ltd, Shenzhen, CHN) 12. The pathological examination of resected tissue was completed in pathology department of Sichuan Cancer Hospital.

In Sichuan Provincial People's Hospital, the SOMATOM Force CT (SIEMENS Healthineers Co., Ltd, Erlangen, GER) was used to detect pulmonary nodules. The platelet features were detected with automatic hemacytology analyzer (Sysmex, Shanghai, Ltd, Shanghai, CHN). The pathological examination was completed in pathology department of Sichuan Provincial People's Hospital.

To make sure the reliability of all the measures, all the measurements were finished according to the standard operation procedure (SOP). The internal quality control (IQC) for LDCT, automatic hemacytology analyzer, and pathology examination needed to be acceptable. And the results of personnel parallel experiments for all the departments also needed to be acceptable. Moreover, the external quality assessment (EQA) results of clinical laboratory and pathology department needed to be 91 to 100 points from 2018 to 2021.

### 2.4 Model Construction

A prediction model was constructed using eXtreme Gradient Boosting (XGBoost). XGBoost is a machine learning technique, which generates a series of decision trees in a gradient boosting manner. In this research, a total of 24 features were entered into multi-tree XGBoost, including gender, smoking status, nodule characteristics on LDCT scan, anamnesis, personal history, family history, and platelet features. And all features were ranked by this model based on the values of their importance. Subsequently, the top 5 features were added to the XGBoost model. After adjusting the features, the final model was created with the importance scores re-calculating.

### 2.5 Model Performance and Validation

The receiver operating characteristic (ROC) curve was used to estimate the performance of the model, which reflected the sensitivity (Sens), specificity (Spec), accuracy (ACC), and the area under the curve (AUC) in the plot. Prediction model performance was compared with other clinical models by using measures of reclassification (NRI and IDI) 13. NRI was referred to the movement of patients from benign nodules to malignant nodules based on different models. IDI was calculated to measure the discrimination, which referred to the ability of a model correctly distinguishing between benign and malignant group. In this research, net benefit (NB), assessed the increase in the proportion of patients with pulmonary nodules receiving the appropriate surgery, after estimation by the predictive models 14. The net benefit of each model over a whole range of threshold probabilities of outcome was graphically displayed as a decision curve.

### 2.6 Statistical Analysis

All analyses were performed by using the SPSS 22.0 software (SPSS Inc., Chicago, IL) and R 4.0.4 (Version 1.74) (R Foundation for Statistical Computing, http://www.R-project.org). The data from malignant patients were compared to the benign patients using the Wilcoxon test. Thus, p<0.05 were considered significant with two sided, and values were indicated in medians. The construction, performance and validation of the model were implemented under R either.

## 3. Results

### 3.1 The demographics and characteristics of participants

After excluding the patients who were lack of pathology results or leaving hospital without surgery, a total of 419 patients with pulmonary nodules were enrolled in the development group finally, which included 295 in training cohort and 124 in testing cohort respectively. The basic clinical information and CT information were summarized in Table 1. The age, gender and pathological stage were adjusted between two cohorts.

### 3.2 Construction of the SCHC model

**Figure 2:**
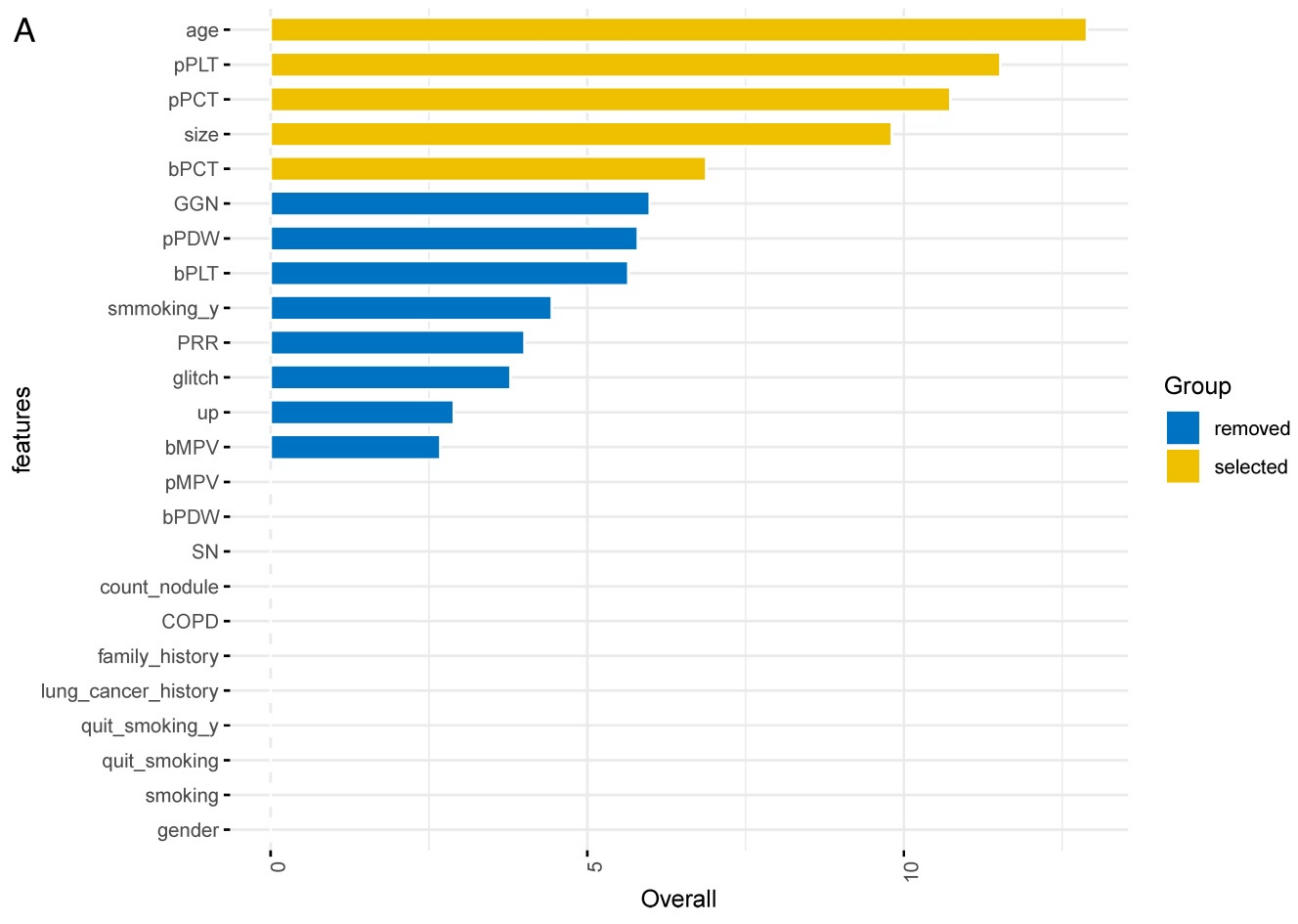

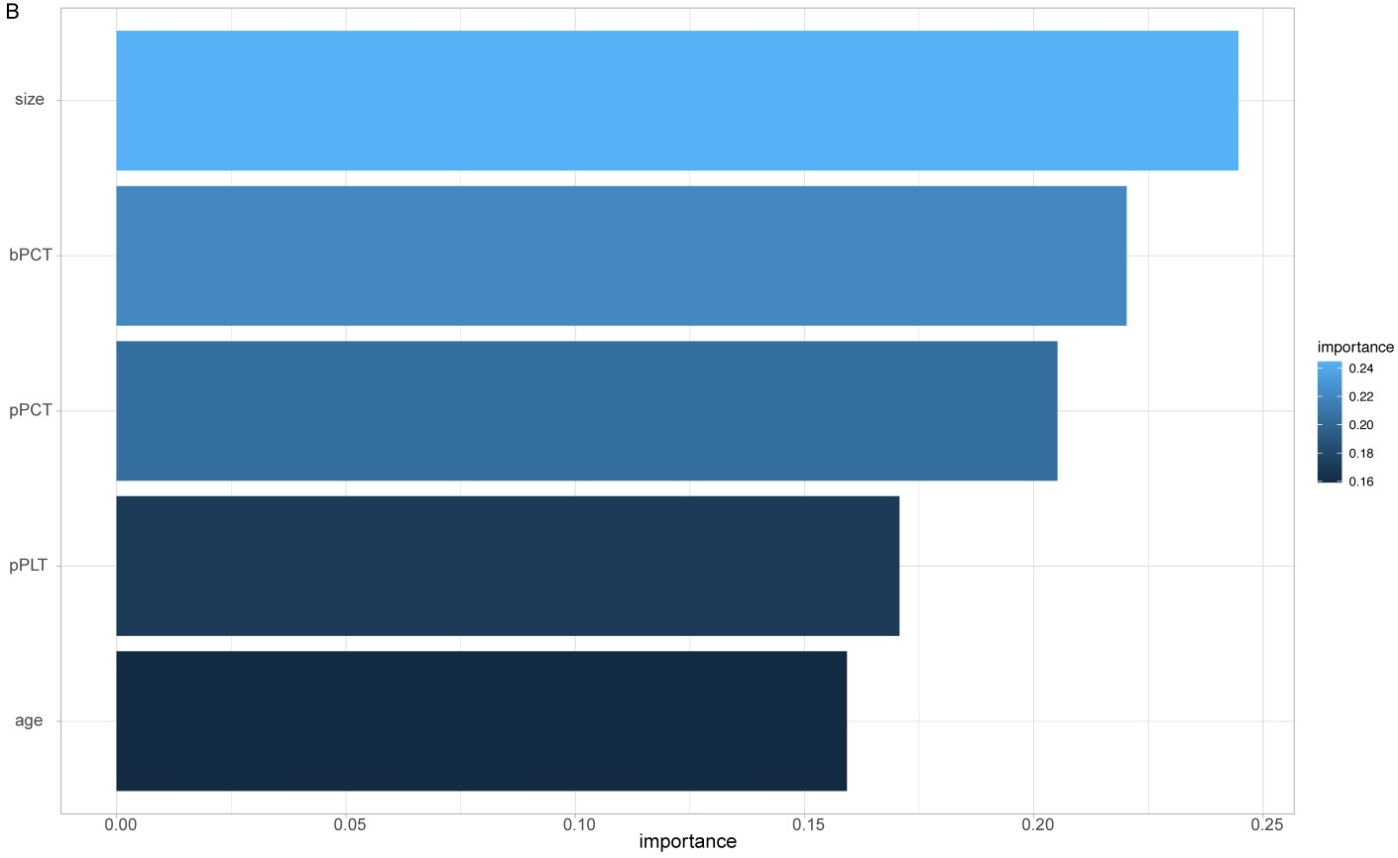
ALL the feature importance scores for the XGBoost model (A). Feature importance scores for the final model (B). Size: the largest diameter of the nodules in millimeter detected by LDCT; bPLT: platelet counts in whole blood sample; bMPV: mean platelet volume in whole blood sample; bPDW: platelet distribution width in whole blood sample; bPCT: plateletcrit in whole blood sample; pPLT: platelet counts in PRP samples; pMPV: mean platelet volume in PRP samples; pPDW: platelet distribution width in PRP sample; pPCT: plateletcrit in PRP sample; GGN: the nodule is ground glass/nonsolid; smoking_y: the years of smoking; glitch: the edge of nodule has spicules; up: the nodule is located in an upper lobe; SN: the nodule is solid; COPD: the patient has a history of COPD; family_history: the patient has a family history of lung cancer; lung cancer history: the patient has a history of extrathoracic cancer that was diagnosed 5 years ago; quit_smoking_y: years since the patient has been quitting smoking; quit_smoking: whether the patient has quit smoking; smoking: whether the patient has smoked.

The importance of all 24 features was ranked by XGBoosting model (Figure 2A). Subsequently, the adjusted top 5 features were added to the final model called SCHC model, including age, platelet counts in PRP sample (pPLT), plateletcrit in PRP sample (pPCT), nodule size, and plateletcrit in whole blood sample (bPCT) (Figure 2B).

### 3.3 Performance and internal validation of the SCHC model

**Figure 3:**
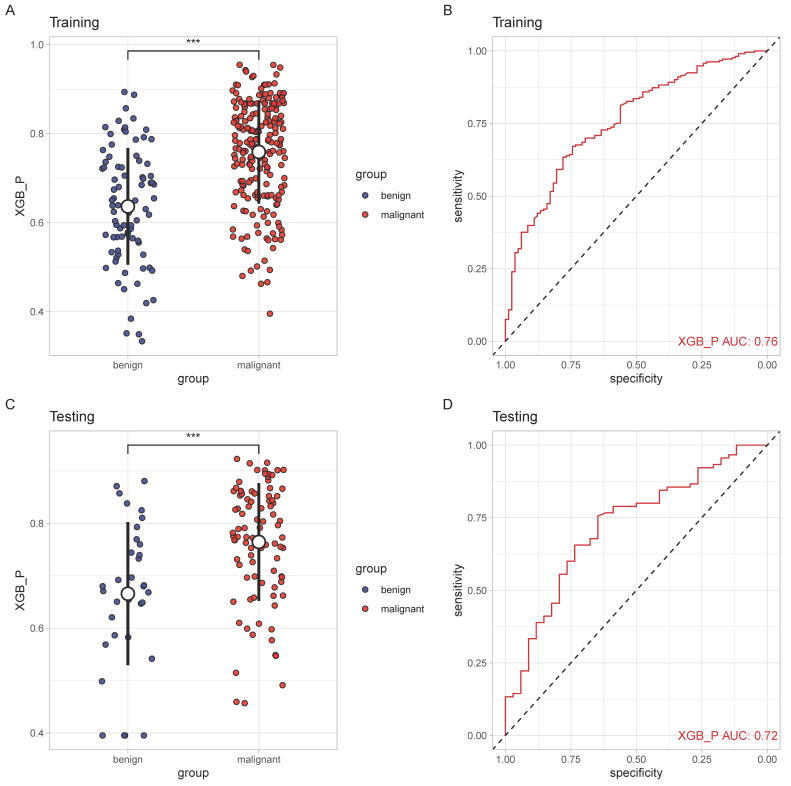
The performance of SCHC model in training and testing cohort. Scatter plots indicating the predictive probabilities calculated by SCHC from the benign and malignant groups of training cohort(A) and testing cohort(C). Reciever operating characteristic curves for performance of SCHC model in training cohort (B) and testing cohort(D). *p < 0.05, **p < 0.01, ***p < 0.001, ****p < 0.0001.

The performance of SCHC model in two cohorts was presented in Figure 3. In two cohorts, SCHC model exhibited a good performance (AUC: 0.76 and 0.72, respectively). The predicted probabilities of malignant set were higher than the benign set in the two cohorts (p<0.001), which indicated the model could differentiate the malignant nodules from the benign nodules.

**Figure 4:**
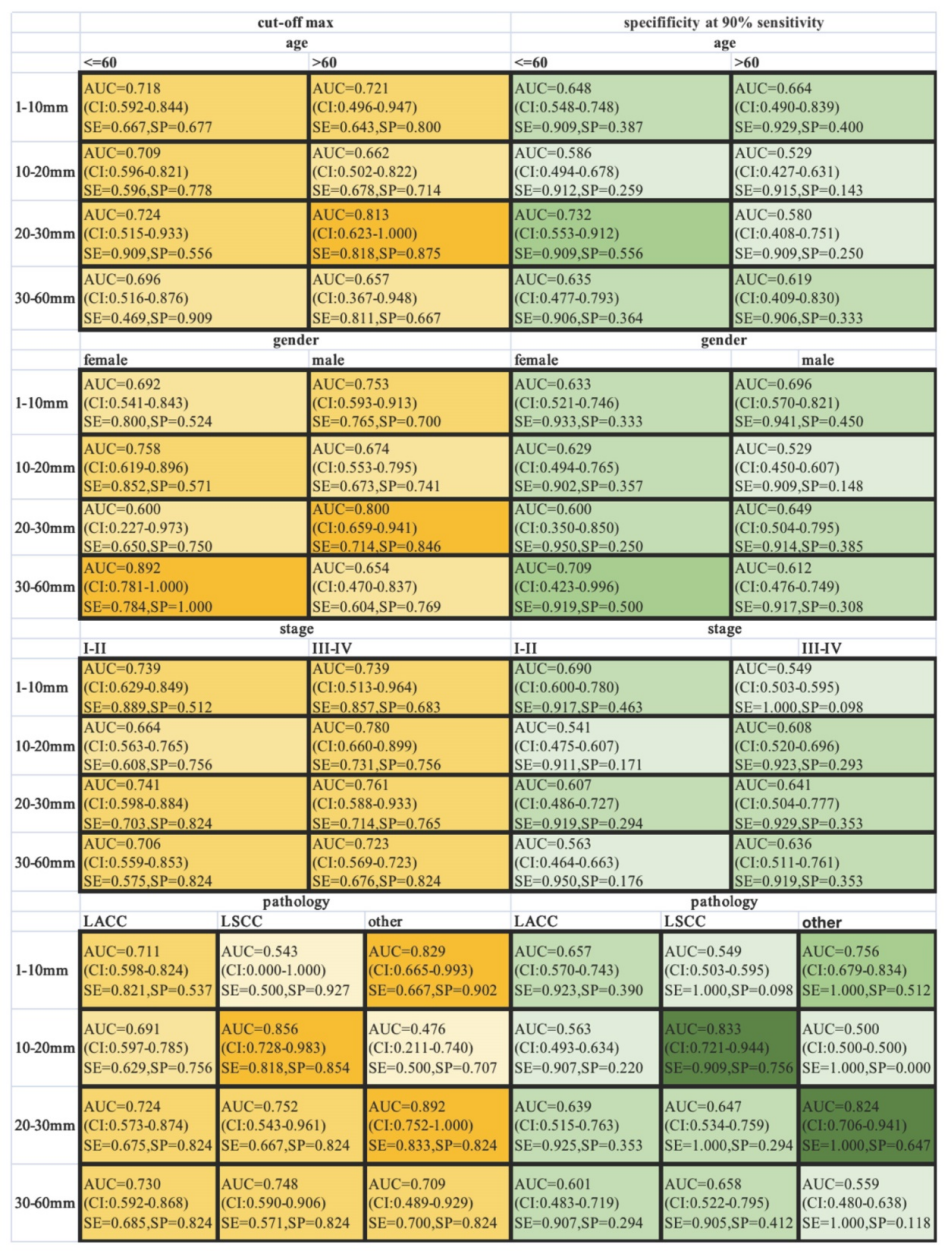
Classification performance in different subgroup patients across different nodule size ranges. The ROC-AUC, specificity and sensitivity (AUC at max, yellow, left; at 90% sensitivity, green, right) were given for the different age, gender, stage and pathology. The vertical axis was different range of nodule size in millimeter. The color intensity was related to the AUC values (AUC at max, yellow) and specificity (at 90% sensitivity, green). SE: sensitivity; SP: specificity.

ROC-AUCs, specificities and sensitivities were calculated for different-size nodules, when the AUC was maximized, or the sensitivity was held at a performance of 90 % (Figure 4). SCHC model performed well on the individuals who were older than 60 with nodules in the 20-30 mm range, achieving 87.5% specificity and 81.8% sensitivity with highest AUC. The SCHC model also performed well on male with same size nodules. And the performance on the LSCC patients with nodules in the 10-20 mm range was particularly well (AUC=0.856, with the 81.8% specificity and 85.4% sensitivity). As the small size nodules were hard to distinguish, when the sensitivity was held at a performance of 90 %, the specificity of SCCH model were at the range from 9.8% to 51.2%. In the clinical applications, the thresholds could be chosen from the different goals in different individuals.

### 3.4 Comparison among SCHC model with other models

**Figure 5:**
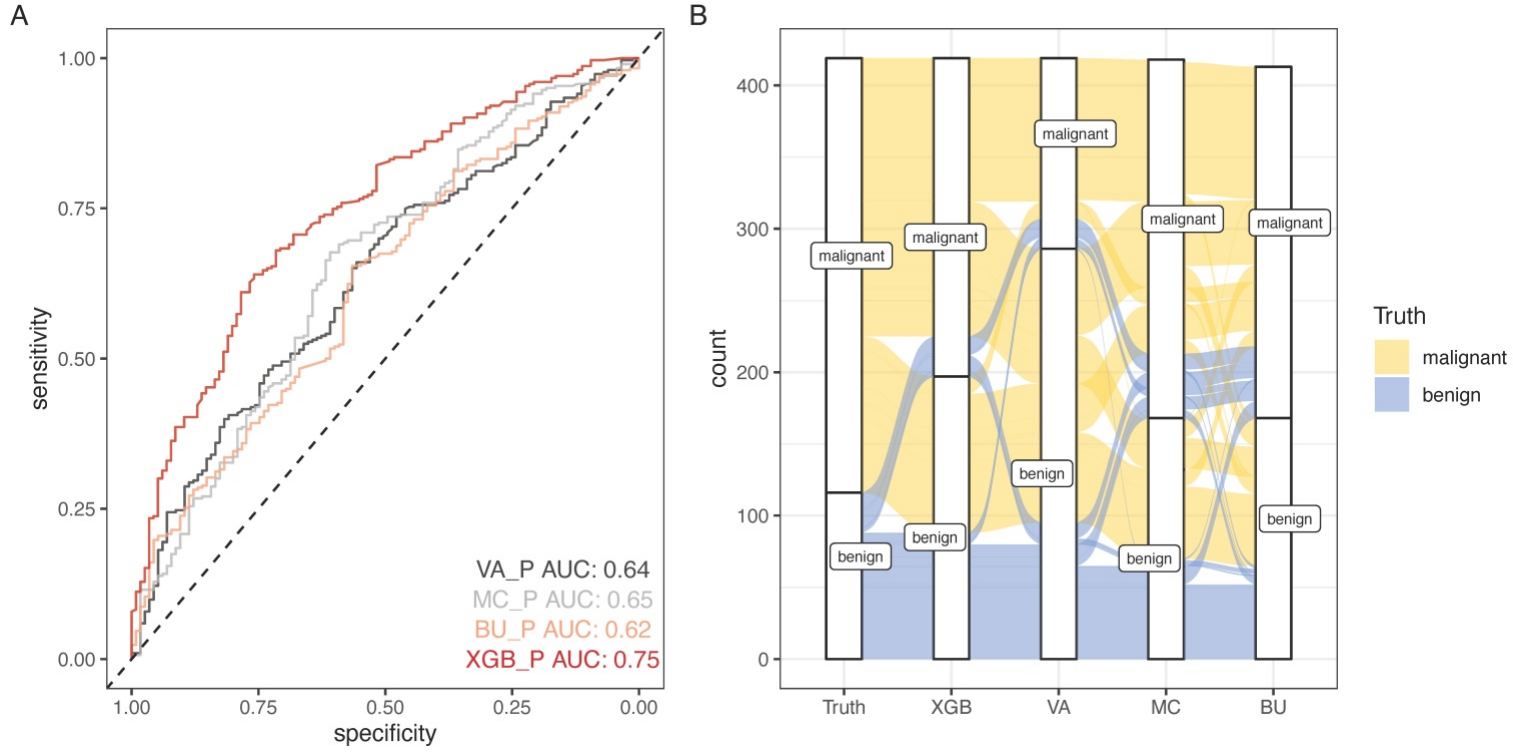

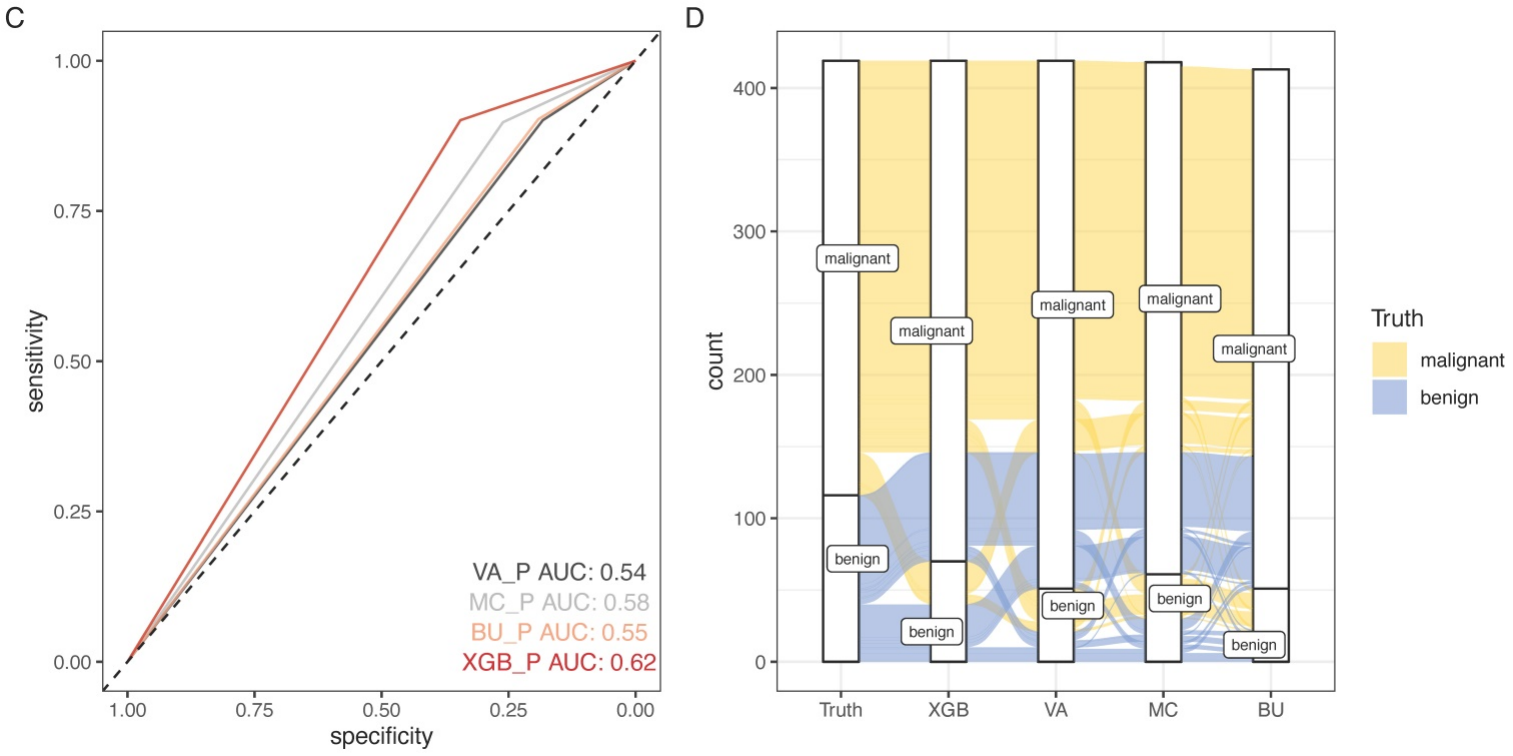

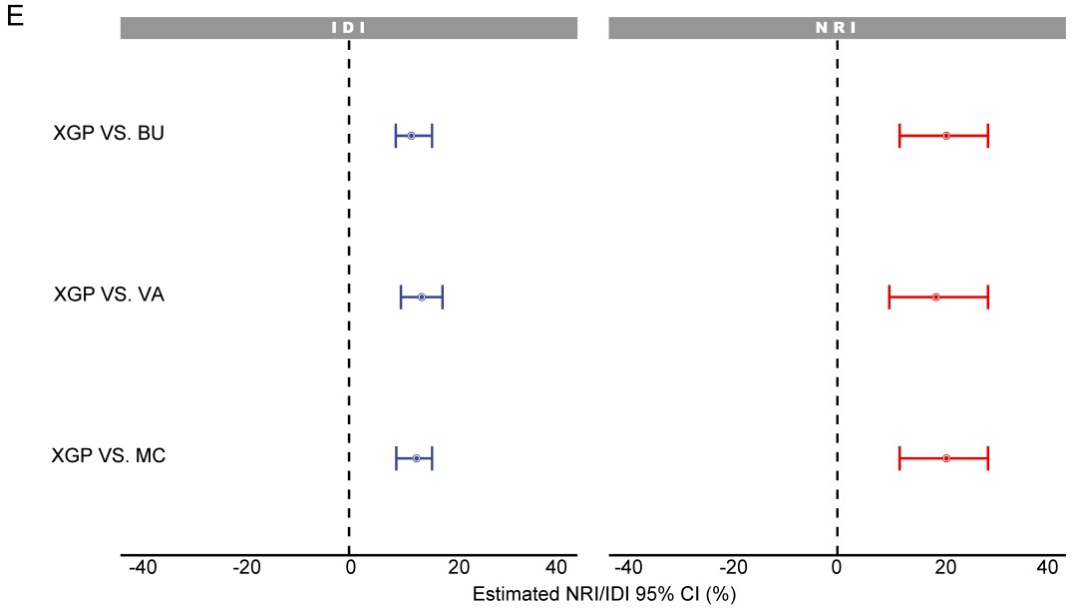

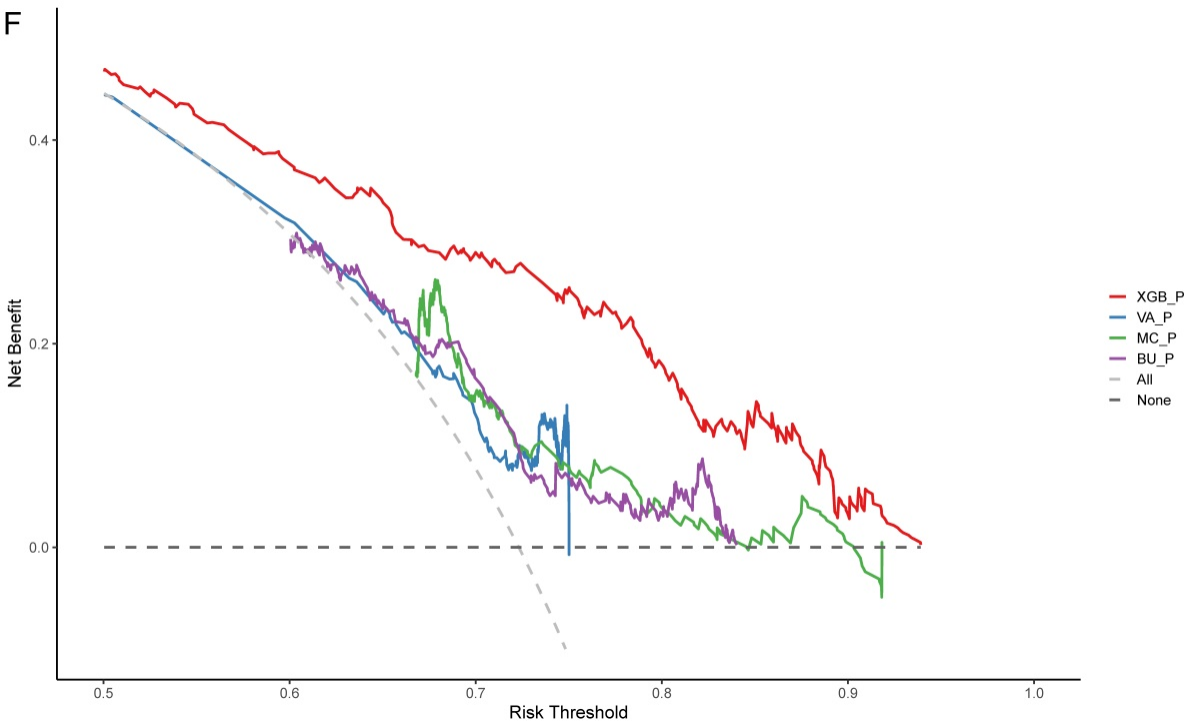

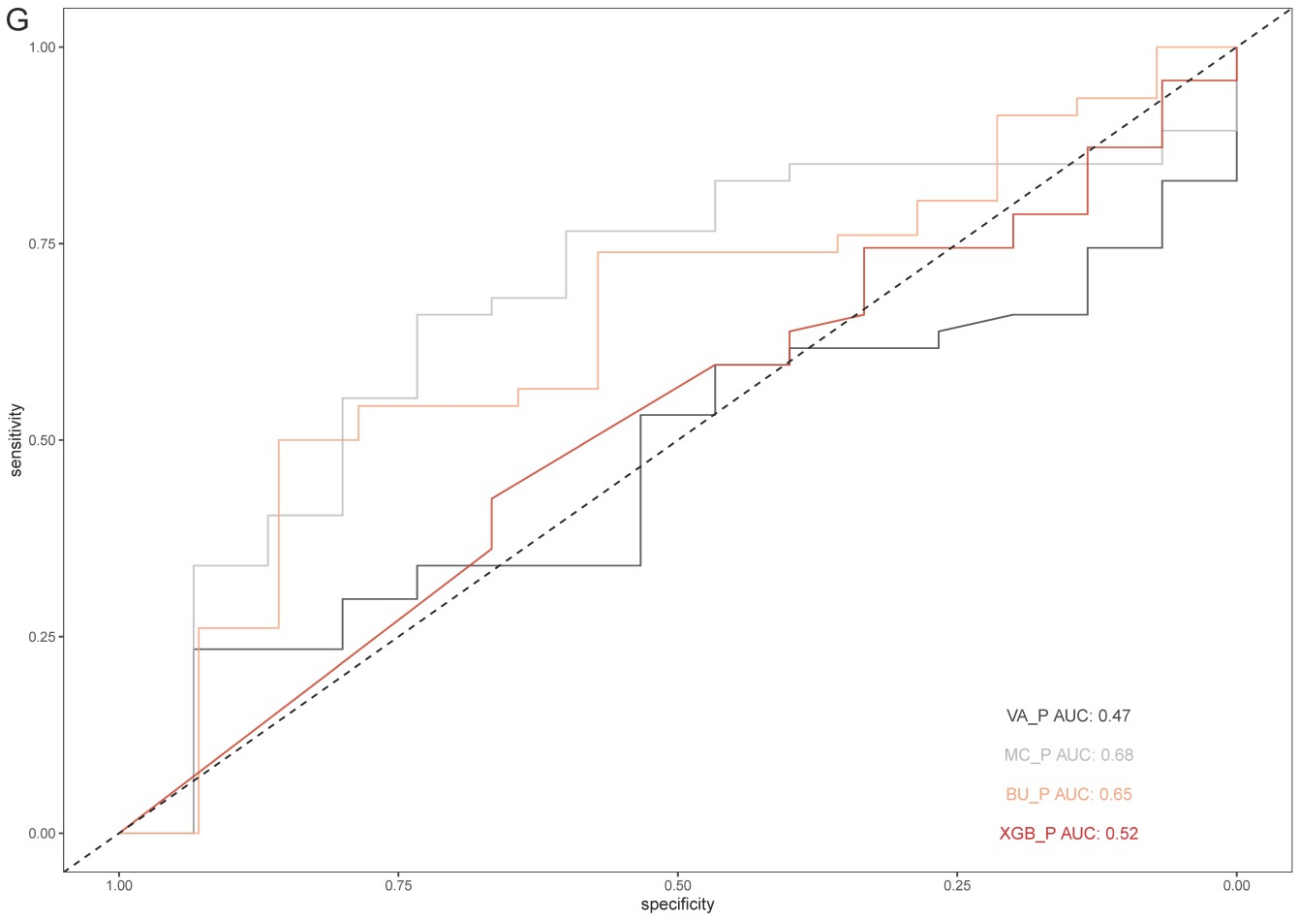
The performance of SCHC model in development cohort compared with other three models. Reciever operating characteristic curves for performance of the model, when the AUC was held at a performance of the max (A), and the sensitivity was held at a performance of 90 % (C). Alluvial diagrams indicating the misclassification for all the models, when the AUC was held at a performance of the max (B), and the sensitivity was held at a performance of 90 % (D). VA_P: the probabilities calculated using VA model; MC_P: the probabilities calculated using MC model; BU_P: the probabilities calculated using BU model; XGB_P: the probabilities calculated using SCHC model. Integrated discrimination improvement for discrimination (IDI), net reclassification improvement (NRI) in the development cohort for SCHC model and other three models (E). Decision curve shown for all models across all threshold probabilities (F). Receiver operating characteristic curves for performance of the models in validation cohort (G).

SCHC model was performed and compared with other clinical models on all participants in development group (Figure 5). Alluvial diagrams indicating the misclassification for all the models were plotted in Figure 5B and Figure 5D. SCHC model misclassified approximately 100 of malignant individuals as benign individuals, which was same as MC and BU models. But the VA model misclassified almost 200 of malignant individuals as benign individuals, whose misdiagnosis rate was higher than the other three models. Instead, the miss rate of VA model was lower than the other three models. SCHC model misclassified approximately 20 of malignant individuals as benign individuals, while the MC and BU model misclassified about 30 of malignant individuals as benign individuals. SCHC model could be seen as the most extreme model in misclassifying the malignant and benign nodules. Compared with the MC model, SCHC had significant improvement in reclassification as assessed by the IDI (0.1274, 95%CI: 0.0909 - 0.1638), and NRI (0.2053, 95%CI: 0.1177 - 0.2928). SCHC also showed a better reclassification ability than VA and BU model with significant NRI (0.1906, 95%CI: 0.0955 - 0.2857, 0.2053; 95%CI: 0.1177 - 0.2928, respectively) and IDI (0.1371, 95%CI: 0.0971 - 0.1772; 0.1235, 95%CI: 0.0883 - 0.1586, respectively) (Figure 5E). The decision curves plotted the net benefits achieved by making decisions based on the four models (Figure 5F). The decision curve displayed that SCHC model with the highest net benefit at all threshold probabilities, which meant SCHC model had the highest clinical value. Thus, no matter what probability threshold was chosen by clinicians, the SCHC model could bring the patients more benefits than the other three clinical models.

### 3.5 External Validation

Based on the results above, SCHC model was further assessed in external validation cohort. 15 benign and 47 malignant participants were included in the external validation cohort. The basic information was presented in Table S1. The accuracy of SCHC model presented limited in the external cohort with an AUC of 0.52 (95% CI, 0.354 to 0.684), which was similar with VA model (AUC=0.47, 95% CI: 0.326 to 0.651), while the MC and BU model outperformed with the AUC of 0.68 (95% CI, 0.520 to 0.833) and 0.65 (95% CI, 0.479 to 0.813) respectively (Figure 5G).

### 3.6 Web tool based on SCHC model

**Figure 6:**
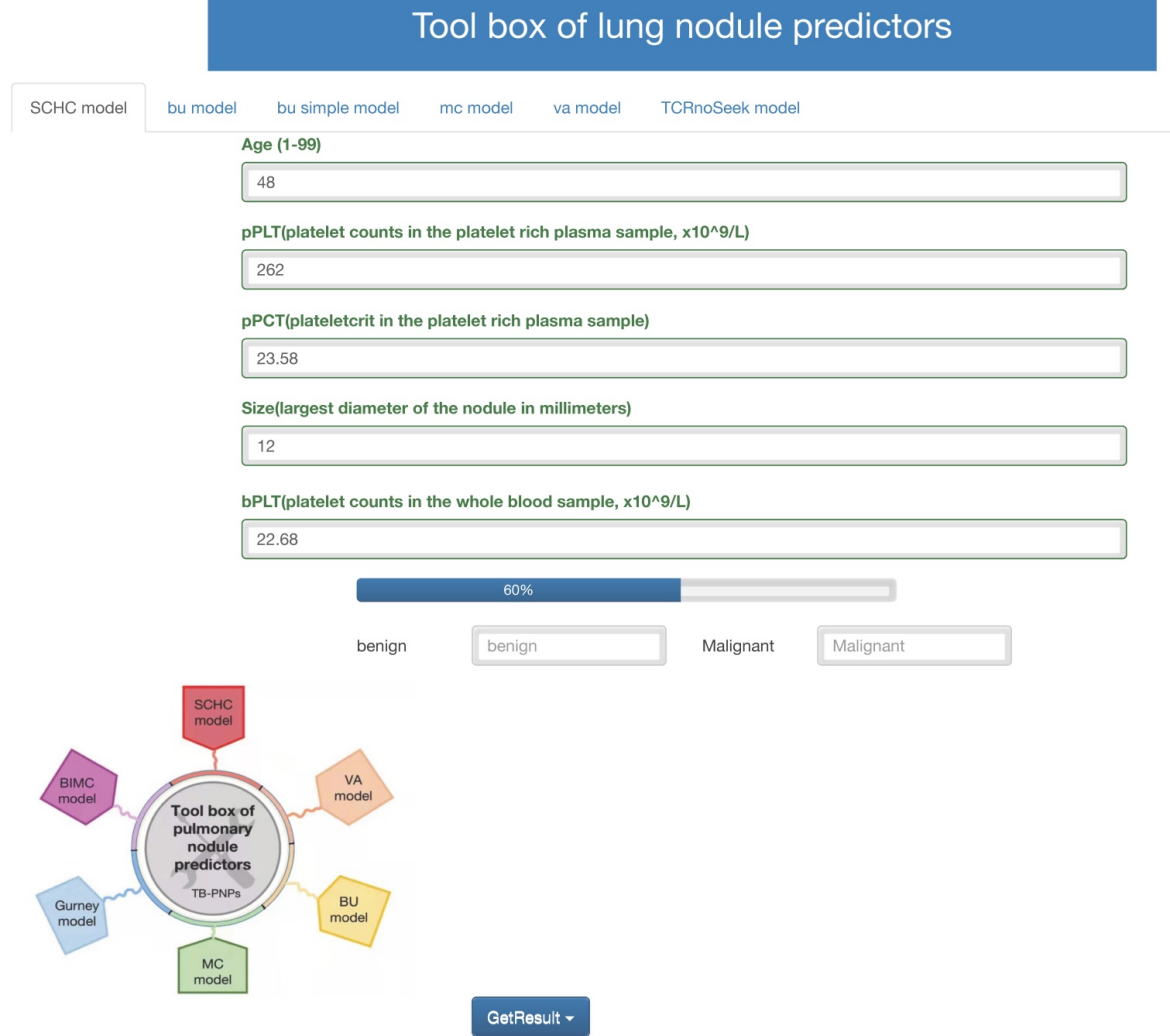
Web tool of the SCCH model for calculating the probability of malignant pulmonary nodules.

Accessing http://i.uestc.edu.cn/SCHC in a web browser displayed directly the main page of SCHC model, where Age, pPLT, pPCT, size, bPCT could be input. By clicking the “Get-Result” button, the probability of malignancy would display with the blue bar, and “malignant” would output if the probability exceeded the threshold. As shown in Fig. 6, users would be allowed to choose SCCH, BU, MC and VA model at the top of the Web page. The web tool would also be updated in the future work along with our follow-up work.

## 4. Discussion

This prospective and observational real-world study provided a reliable model, which could distinguish the benign nodules from malignant nodules based on platelet features, age and nodules size. Compared with other common models, the incremental improvements in AUC, NRI, IDI and NB indicated an accurate diagnostic value of our SCHC model.

As the lung cancer has become an important public health problem, the LDCT was a widely used screening test for the suspicious pulmonary nodules. A large part of the suspicious pulmonary nodules was proved to be false positive, which might associate with an unnecessary surgery. In order to help clinicians improve the predictive accuracy for the pulmonary nodules, a number of clinical models were published.

The clinical models, like VA model, MC model and BU model, were widely cited in previous researches. The VA model was developed with the data from Department of Veterans Affairs (VA), which calculated the probabilities of malignancy in patients with solid pulmonary nodules (SPNs) using the patients' smoking history, age and nodule size. The accuracy of VA model presented well in the internal cohort with an AUC of the ROC curve of 0.79 (95% CI, 0.74 to 0.84), but not well in the external researches 15, 16, which was same in this research. The MC model was proposed by Swensen 7 at the Mayo Clinic, which expressed the probabilities of malignant nodules as a function of the variables including age, smoking history, cancer history and characteristics of the nodules. But the MC model performed not much stable in other validation researches, with the AUC range from 0.60 to 0.89 10, 17-19. The BU model also named Brock model was developed from the Pan-Canadian Early Detection of Lung Cancer Study, which included two sets of models: a parsimonious model and a full model. The BU model calculated the probabilities using multivariable logistic regression based on the variables including age, gender, family history of lung cancer, presence of COPD, nodule size, nodule location, nodule counts, and nodule characteristics. In the other external validation researches, the BU model performed excellent discrimination, with an AUC greater than 0.80 18-21. Most of the clinical models were retrospective researches and set up with malignant and healthy individuals, so the three models showed limited values in this research. And SCHC model was set up with malignant and benign nodule patients, so it performed better than the other models in our research.

Except for the clinical models, the liquid biopsy has also been hot in the researches of diagnosing malignancy. Especially in lung cancer, multiple biomarkers like DNA methylation and serum microRNAs have been proved great diagnostic values with high sensitivities and specificities 15, 22. But the detection of DNA methylation or serum microRNAs is complicated and costly, which is not suitable for the widely screening. Moreover, the variables of our model including age, platelet features and nodule size are all easy to estimate. The age and nodule size have been reported in other models, which reveals the two factors taking roles in the prediction of malignant nodules. And the platelet features are new factors applied in predicting the malignancy. Platelets are the second most components of blood, which not only play the key role in hemostasis, but also in cancer progression. As the concepts of tumor educated platelets (TEPs) were presented, it have been proven that the tumor cells could activate the platelets, and then the activated platelets release molecules to impact the tumor cells in turn 23-25. And platelet features are accessible by hematology analyzer, so the detection could be automatic, standard, recurring and low-cost. In our previous research, we set up a multivariate logistic regression model to diagnose lung cancer based on the platelet features, with the AUC of 0.92 for the training cohort and 0.79 for the testing cohort, which means platelets could be noninvasive biomarkers for a new liquid biopsy of classifying the pulmonary nodules. This research also indicated that common laboratory results also have the potential in diagnosing cancers. The clinical laboratory results include clinical biochemistry, hematology and microbiology results, which offer complexity and a variety of information of patients. So, combination the common laboratory results could be used in diagnosing different diseases, not only cancers, which also provide a supererogation of the clinical laboratory science.

This research was a prospective and observational study, which could provide more reliable data to influence the predicted probabilities. Recently, real-world studies (RWS) are gaining increasing attention since the United States Food and Drug Administration (FDA) and the Chinese Food and Drug Administration (CFDA) presented that RWS could be used in etiology, diagnosis, therapy and prognosis 33. Machine learning has been used in real-world data for numerous disease diagnoses. The XGBoost model is a machine learning method used in many medical prediction models for the high precision prediction 26-29. As reported in the previous researches, the XGBoost algorithm always achieved more excellent performance compared with other algorithm such as Logistic regression, Bayesian analysis and so on 30-32. XGBoost could provide the importance score of each variable, and then the top 5 of the variables were selected into the final model. And compared with VA, MC, and BU model, the XGBoost model had best prediction effect, which also could bring more benefits to the patients with pulmonary nodules.

Furthermore, a web page was used to display the SCHC model, and totally free to support both patients and clinicians. The input included the platelet features which were accessible and inexpensive, while the output contained the probability values. This web tool also first integrated the VA, MC, and BU model together, which was easy to calculate the probability of malignancy requiring no programming knowledge. While choosing a strategy for evaluating patients with lung nodules, the clinicians could consider the probability calculated with the web tool. Those nodules with low-risk probabilities, the “benign” outputs could allow the nodules to be surveyed by serial imaging.

There were still some limitations for the SCHC model. In order to validate our model, we further collected an external cohort from Sichuan Provincial People's Hospital. But the performance of the SCHC model was not good. The SCHC model was constructed with platelet features in PRP samples, which might be influenced by the different centrifuges, laboratory environment, and manipulators. The platelets were easily activated by external force, and the activated platelets were more likely to aggregate, relating to the incorrect results of plateletcrit. This might be the reason for the poor performance in the external validation. The small size of validation data might also contribute to the limitation of the SCHC model. In the future work, we would increase the validation sample size, and construct a simpler and more constant operating manual to minimize the influence in the different system.

## 5. Conclusion

We have developed and validated a predictive model with good performance characteristics based on the real-world data for identifying malignant nodules from benign nodules more accurately. And SCHC model used the age, platelet features, and nodule size, which could be easily implemented and would not take additional cost of patients. Thus, SCHC model could be widely applied in the screening of patients with pulmonary nodules, and might reduce the invasive procedures by recognizing benign nodules to surveillance. We developed a user-friendly web tool that integrated SCHC model and common clinical models together making a broadly availability (http://i.uestc.edu.cn/SCHC). And this research also provided evidence for the supererogatory uses of common laboratory results.

## Supplementary Material

Supplementary methods, figures and tables.

## Figures and Tables

**Table 1:** The demographics and characteristics of all participants

	Traning			Testing		
	**benign**	**malignant**	**p.overall**	**benign**	**malignant**	**p.overall**
	82 (27.8%)	213 (72.2%)		34 (27.4%)	90 (72.6%)	
gender:			0.215			0.095
Female	32 (39.0%)	102 (47.9%)		11 (32.4%)	46 (51.1%)	
Male	50 (61.0%)	111 (52.1%)		23 (67.6%)	44 (48.9%)	
up:			0.096			0.746
down	41 (50.0%)	82 (38.5%)		15 (44.1%)	35 (38.9%)	
up	41 (50.0%)	131 (61.5%)		19 (55.9%)	55 (61.1%)	
GGN:			0.276			0.287
GGN	10 (12.2%)	39 (18.3%)		1 (2.94%)	10 (11.1%)	
non-GGN	72 (87.8%)	174 (81.7%)		33 (97.1%)	80 (88.9%)	
glitch:			0.024			0.109
glitch	14 (17.1%)	66 (31.0%)		6 (17.6%)	31 (34.4%)	
non-glitch	68 (82.9%)	147 (69.0%)		28 (82.4%)	59 (65.6%)	
age:			0.091			0.014
<=60	52 (63.4%)	110 (51.6%)		26 (76.5%)	45 (50.0%)	
>60	30 (36.6%)	103 (48.4%)		8 (23.5%)	45 (50.0%)	
stage:			<0.001			<0.001
benign	82 (100%)	0 (0.00%)		34 (100%)	0 (0.00%)	
I-II	0 (0.00%)	135 (63.4%)		0 (0.00%)	57 (63.3%)	
III-IV	0 (0.00%)	59 (27.7%)		0 (0.00%)	25 (27.8%)	
NS	0 (0.00%)	19 (8.92%)		0 (0.00%)	8 (8.89%)	
smoking:			0.888			1.000
Ever/current	29 (35.4%)	79 (37.1%)		13 (38.2%)	35 (38.9%)	
Never	53 (64.6%)	134 (62.9%)		21 (61.8%)	55 (61.1%)	
size:			1.000			0.620
<=3cm	46 (56.1%)	118 (55.4%)		22 (64.7%)	52 (57.8%)	
>3cm	36 (43.9%)	95 (44.6%)		12 (35.3%)	38 (42.2%)	
