# Supplementary material for: A new classifier constructed with platelet features for malignant and benign pulmonary nodules based on prospective real-world data
Source: J Cancer. 2022 May 9;13(8):2515–27. doi: 10.7150/jca.67428 (PMC9174863; doi:10.7150/jca.67428)
Supplement: Supplementary file 1 — Supplementary methods, figures and tables. [file jcav13p2515s1.pdf]

Supplements

1. There were total of 62 individuals included in the external validation cohort. And the basic information of external validation cohort were presented in the Table S1.

Table S1 The basic information of external validation cohort

	**benign**<br>**N=15**	**malignancy**<br>**N=47**	**p.overall**
gender:			0.049
F	6 (40.0%)	34 (72.3%)	
M	9 (60.0%)	13 (27.7%)	
pathology:			<0.001
benign	15 (100.00%)	5 (10.6%)	
LACC	0 (0.00%)	35 (74.5%)	
LACC	0 (0.00%)	2 (4.26%)	
NSCLC	0 (0.00%)	2 (4.26%)	
other_malignant	0 (0.00%)	3 (6.38%)	
up:			0.051
down/middle	12 (80.0%)	22 (46.8%)	
up	3 (20.0%)	25 (53.2%)	
GGN:			0.076
GGN	5 (33.3%)	30 (63.8%)	
non-GGN	10 (66.7%)	17 (36.2%)	
glitch:			0.052
glitch	0 (0.00%)	11 (23.4%)	
non-glitch	15 (100%)	36 (76.6%)	
age:			0.985
<=60	9 (60.0%)	26 (55.3%)	
>60	6 (40.0%)	21 (44.7%)	
stage:			<0.001

	benign N=15	malignancy N=47	p.overall
	0 (0.00%)	8 (17.0%)	
benign	15 (100%)	0 (0.00%)	
I	0 (0.00%)	36 (76.6%)	
II	0 (0.00%)	3 (6.38%)	
smoking:			0.016
ever/current	5 (33.3%)	3 (6.38%)	
never	10 (66.7%)	44 (93.6%)	
size:			0.667
<=3cm	14 (93.3%)	40 (85.1%)	
>3cm	1 (6.67%)	7 (14.9%)	

2. **Comparison among SCHC model with other models**

In order to compare the SCHC model with other models including classical and new moedels, we randomly selected 97 individuals from the development group, including 25 benign and 72 malignant patients. The basic information of the random cohort were displayed in the **Table S2.** A total of 5 models were used to calculated the parobabilities, including VA model, MC model, BU model, TG model, PU model and SCHC model. And the probabilities calculated by 6 models were compared in **Table S2**. And the ROC curves were draw to evaluated the diagnostic value. As shown in **Table S3** and **Figure S1**, the SCHC model exceeded than the other 5 models with the AUC at 0.82.

The information of new moedels(TG model, PU model) was as follows:

The Tongji model(TG model) were developed by the Tongji University in 2017, a primary cohort was consist of 1798 patients, and the formula to calculated the malignant probability was as follows:

$P = e^x/(1+e^x)$,

X=-5.4175 + 0.8149×LgCEA+ 1.0447×Nodule diameter(mm) + 2.5978×Cancer history + 0.0518×Age + 1.7166×Spiculation+0.3986×Pleural indentation-2.2549×calcification.

In their original study, they compared the model with MC model, VA model , BU model and Beijing university. And their results showed that the AUC of Tongji model (0.85, 95%CI: 0.83-0.85)was higher than the other 4 models (0.8,95%CI: 0.77-0.82; 0.65,95%CI:0.62-0.68; 0.83,95%CI: 0.8-0.86;0.8,95%CI: 0.78-0.83).(DOI: 10.1002/jso.24704)

And the Peking University model (PU model) was developed by the Peking University in 2011, including 371 individuals. And the formula to calculated the malignant probability was as follows:

$P=e^x/(1+e^x)$,

X=-4.496+(0.07×Age)+(0.676×diameter)+(0.736×spiculation)+(1.267×family history of cancer) - (1.615×calcification) - (1.408×border).

As they reported , the AUC of Peking University model was 0.888, which also exceeded than MC model (AUC 0.747) and VA model(AUC 0.707). (doi: 10.1016/j.cllc.2011.06.005)

Table S2 The basic information of appendix cohort

		benign	malignancy	p
**Total**		25	72	
**gender**	**female**	**9**	**37**	0.246
	**male**	16	35	
**age**	**>60**	6	38	0.019
	**<=60**	19	34	
**pathology**	**benign**	25		0.000
	**LACC**		59	
	**NSCLC**		5	
	**LSCC**		6	
	**other_malignant**		2	
**stage**	**benign**	25		
	**I**		41	
	**II**		10	
**size**	**<=3cm**	24	56	0.063
	**>3cm**	1	16	
**VA_P**		0.9955	0.9983	0.014
**MC_P**		0.0097	0.0375	0.000
**BU_P**		0.0372	0.2114	0.000
**XGB_P**		0.6224	0.7587	0.000
**TG_P**		0.9993	1	0.001
**PU_P**		0.9918	0.9999	0.000

Table S3 The ROC results of 6models in appendix cohort

Index	value-VA	value-MC	value-BU	value-XGB	value-TG	value-PU
**AUC(%)**	66.64	76.00	75.56	81.89	72.23	74.44
**Best Cut-off Value**	1.00	0.03	0.10	0.74	1.00	1.00
**Sensitivity**	75.00	58.33	72.22	61.11	82.86	83.33
**Specificity**	60.00	88.00	80.00	92.00	60.00	64.00
**Negative Predictive Value**	45.45	42.31	50.00	45.10	55.56	57.14
**Positive Predictive Value**	84.38	93.33	91.23	95.65	85.29	86.96
**True Positive Rate**	75.00	58.33	72.22	61.11	82.86	83.33
**False Positive Rate**	40.00	12.00	20.00	8.00	40.00	36.00
**True Negatice Rate**	60.00	88.00	80.00	92.00	60.00	64.00
**False Negative Rate**	25.00	41.67	27.78	38.89	17.14	16.67
**False Discovery Rate**	15.63	6.67	8.77	4.35	14.71	13.04
**Accuracy**	71.13	65.98	74.23	69.07	76.84	78.35
**Precision**	84.38	93.33	91.23	95.65	85.29	86.96

**Youden Index**	135.00	146.33	152.22	153.11	142.86	147.33

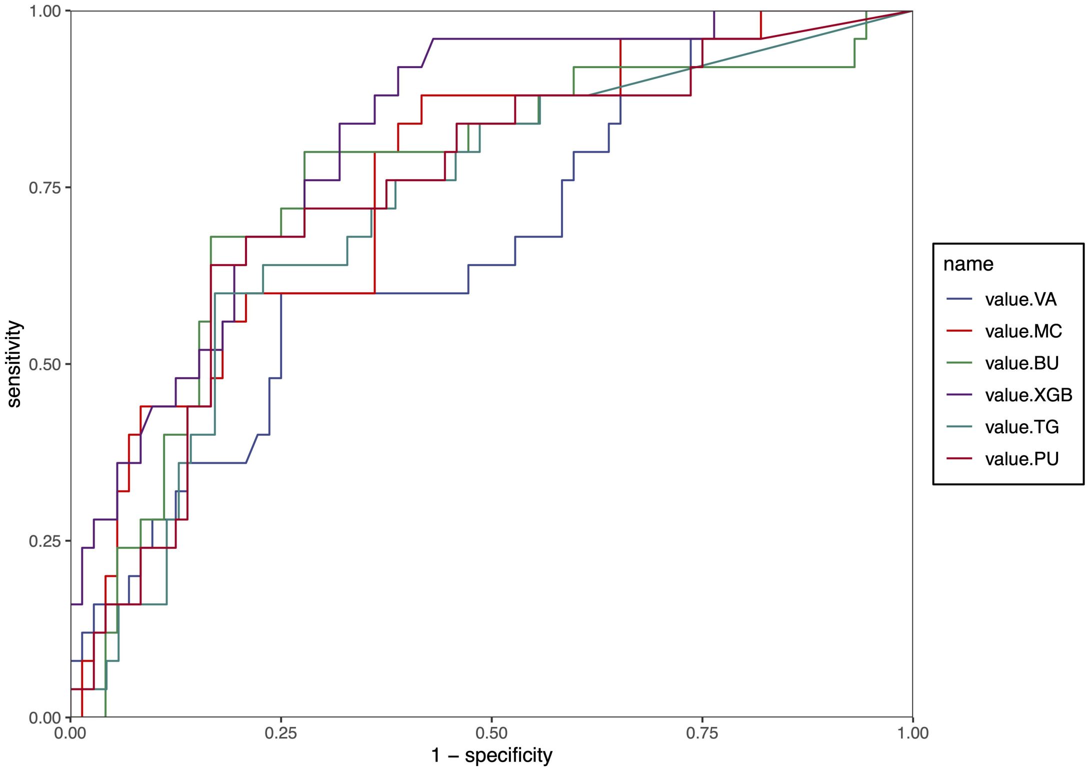

Figure S1 The ROC of 6 models in the appendix cohort
